# TiO_2_/Cu_2_O Heterojunction with Ultrafine Cu_2_O Dispersion and Enhanced Performance for Solar-Driven Hydrogen Production: A Low-Temperature, Ambient Pressure, and Common Stabilizing Agent-Free Synthesis Approach

**DOI:** 10.1021/acsomega.5c03805

**Published:** 2025-08-27

**Authors:** Emanuel do Couto-Pessanha, Victor Magno Paiva, Marianne Diniz Rocha Henriques, Eliane D’Elia, Khrissy Aracélly Reis Medeiros, Jordi Llorca, Bojan A. Marinkovic

**Affiliations:** † Department of Chemical and Materials Engineering, 28099 Pontifical Catholic University of Rio de Janeiro (PUC-Rio), 22453-900 Rio de Janeiro, RJ, Brazil; ‡ Institute of Energy Technologies, Department of Chemical Engineering and Barcelona Research Center in Multiscale Science and Engineering, 16767 Universitat Politècnica de Catalunya, EEBE, Eduard Maristany 16, 08019 Barcelona, Spain; § Institute of Chemistry, 28125 Federal University of Rio de Janeiro, UFRJ, 21941-909 Rio de Janeiro, Brazil

## Abstract

The development of efficient catalysts for hydrogen photoproduction is a significant research field, as the demand for clean energy increases. In this context, we have successfully applied an innovative synthesis protocol for TiO_2_/Cu_2_O heterojunctions, designed to be carried out at low temperature and ambient pressure without the need for addition of a special stabilizing agent, offering a novel and efficient approach to fabricating these materials. The TiO_2_/Cu_2_O heterojunction demonstrated a notable increase of over 280 times in photocatalytic hydrogen (H_2_) production under sunlight compared to neat TiO_2_ (8.51 mmol h^–1^ g^–1^ vs. 0.03 mmol h^–1^ g^–1^), maintaining reasonable photostability, with ∼15% reduction in activity after the fifth cycle. Furthermore, the material was characterized by XRPD, scanning electron microscopy (SEM), transmission electron microscopy (TEM), DRS, EIS, and N_2_ physisorption. Interestingly, TEM analysis revealed that the applied synthesis protocol—despite not employing any specific capping or stabilizing agent—successfully achieved an excellent dispersion of ultrasmall Cu_2_O species (<2 nm) on the TiO_2_ support. This remarkable dispersion is responsible for the efficiency of the heterojunction interface and provides a key explanation for the outstanding photocatalytic performance observed. The present study represents a step forward in developing photocatalysts for H_2_ generation by demonstrating the potential of a straightforward, low-temperature, capping or stabilizing agent-free, and environmentally benign synthesis protocol to prepare a TiO_2_/Cu_2_O heterojunction as an efficient catalyst for sustainable H_2_ photoproduction.

## Graphical abstract

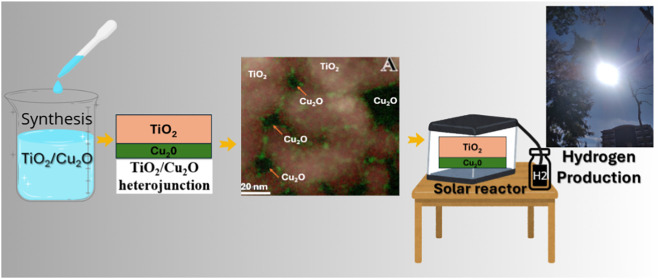

## 1 Introduction

Photocatalytic hydrogen (H_2_) production is seen as a promising alternative for obtaining clean and renewable energy, which is crucial for reducing dependence on fossil fuels and mitigating climate changes.^1–4^ In photocatalytic hydrogen production, it is possible to achieve the direct conversion of the widely and freely available solar energy into chemical energy stored in the H–H bonds of H_2_ using water as the source of H_2_.^5^ Importantly, such direct conversion avoids intermediate steps, which involve energy losses and inefficiencies, both of which are present in the methods currently employed for large-scale H_2_ production.^6^ However, the efficiency of the currently available photocatalyst materials still faces significant challenges. Among these materials, TiO_2_ is widely used due to its high chemical photostability (no photocorrosion), nontoxicity, and relatively low cost.^4,7,8^ However, neat TiO_2_ has drawbacks, such as the high recombination rate of photogenerated electron–hole (e^–^/h^+^) pairs and low absorption of visible light, which limits its efficiency under sunlight since only about 3% of the solar spectrum is in the ultraviolet (UV) region.^9–14^

To overcome these drawbacks, noble metals, such as Au, Pt, and Pd, are commonly used as cocatalysts for H_2_ photoproduction.^15^ Although they exhibit good efficiency in enhancing the photocatalytic activity of TiO_2_,^7,16,17^ catalysts containing these scarce metals face a significant challenge for large-scale applications due to their high costs. Therefore, studies involving TiO_2_-based catalysts containing more abundant metals must be carried out.^15^ In this regard, Cu_2_O is a photocatalytic material that has significant absorption within the visible light range and excellent properties for hydrogen production.^18^ Furthermore, Cu_2_O was referred to as a feasible and low-cost alternative to the aforementioned noble metals for H_2_ photoproduction.^19–21^ However, Cu_2_O alone has poor photostability and is prone to photocorrosion and oxidation. This drawback significantly limits the effectiveness of Cu_2_O alone, especially in long-term photocatalytic applications. The combination of TiO_2_ and Cu_2_O in a heterojunction has been explored as a strategy to achieve synergy between the two phases, resulting in a photocatalyst with increased visible light absorption and lower recombination rate, while stabilizing Cu_2_O phase^21–23^

Trang et al.^24^ demonstrated that the formation of a p-n heterojunction between Cu_2_O and TiO_2_ plays a crucial role in improving photocatalytic performance for H_2_ evolution. In their study, TiO_2_ nanotubes (TNTs) were first synthesized by a hydrothermal method. Afterward, the TNTs were modified with Cu by the impregnation method. The as-prepared TNTs impregnated with Cu_2_O showed increased visible light absorption, resulting in an H_2_ evolution rate of 48 mmol h^–1^ g^–1^ for a 1.5 wt % Cu content, which was a 3.2 times improvement over pure TNTs. This performance enhancement has been attributed to the efficient separation of electron–hole pairs provided by the heterojunction formation. However, the synthesis method proposed by Trang et al.^24^ involves multiple steps, including the use of hydrothermal reactors and subsequent impregnation, making it more complex, time-consuming and energy-intensive, compared to the straightforward approach reported in the present work.

More recently, Xing et al.^21^ investigated the photocatalytic dehydrogenation of ethanol on Cu_2_O/TiO_2_ heterojunctions, using a one-step hydrothermal synthesis strategy to produce nanocomposites with controlled amounts of Cu_2_O. Their results showed that the formed p-n heterojunction supported the separation of electron–hole pairs, resulting in H_2_ production of 24.5 mmol h^–1^ g^–1^ with a Cu_2_O load of 1 wt %, which is about 10 times higher than TiO_2_ alone. Electrochemical impedance spectroscopy (EIS) analysis revealed that the sample with 1 wt % Cu_2_O had lower resistance, suggesting that the heterojunction provided better charge transport and a lower recombination rate.

Finally, Rajendran et al.^25^ presented a one-step sol–gel synthesis strategy to obtain Cu_*x*_O/TiO_2_ photocatalysts with controlled Cu^+^ or Cu^2+^ oxidation states. In that study, the authors highlighted the importance of the copper oxidation state for catalytic efficiency in H_2_ photoproduction. Indeed, the best results were observed for the catalyst labeled TiCu^–1^, which was attributed to the exclusive presence of Cu^+^ species. Catalysts containing mixed oxidation states of copper exhibited about 60–70% of the activity measured for TiCu^–1^. As such, TiCu^–1^ exhibited an H_2_ photoproduction rate of 7.06 mmol h^–1^ g^–1^, which was 35 times higher than that observed for TiO_2_ alone. In addition, it was also demonstrated, through techniques such as diffuse reflectance spectroscopy (DRS) and EIS, that the presence of Cu_*x*_O in TiO_2_ boosts the visible light harvesting, increases the photogenerated current, and enhances photogenerated charge carriers’ lifetime.

Despite the synergy between the TiO_2_ and Cu_2_O phases to produce visible-light-efficient catalysts, the synthesis routes commonly reported in the literature for this material are generally energy-intensive and involve harsh conditions and/or multiple steps to prepare the final product, which hinders its practical application. In this context, the present study reports for the first time, as the authors are aware, a straightforward soft-chemistry synthesis route for TiO_2_/Cu_2_O heterojunction system, capable of dispersing ultrasmall Cu_2_O species (as small as 2 nm) on the nanometric TiO_2_ support. To prepare this photocatalyst, pure nano-TiO_2_, used as a support, was sonicated in deionized water. Following this, copper sulfate, sodium hydroxide, and ascorbic acid (AA) were sequentially added at 50 °C. It is worth mentioning that no common capping or stabilizing agents (whose role is to stabilize nanoparticles’ morphology, dispersion, and surface properties) were used. However, it should be recognized that it has been recently reported that AA, and its byproducts, in alkaline environments not only act as reductants but that it is also plausible to have a role as capping agents.^26^ The final product was then rinsed, dried overnight at room temperature, and labeled as a TiO_2_/Cu_2_O heterojunction.

The goals of this work involve (1) low-temperature synthesis of well-dispersed Cu_2_O species on a nanometric TiO_2_ support by a soft chemical approach, without addition of common capping or stabilizing agents, ensuring a maximized interfaces inside p-n heterojunction; (2) characterization of the structure, morphology, optical, textural, electrochemical, and photoelectrochemical properties of the as-prepared heterojunction; (3) evaluation of the photocatalytic efficiency of the TiO_2_/Cu_2_O heterojunction in H_2_ production under solar light; and (4) evaluation of the cycling stability of the photocatalyst.

## 2 Experimental Methods

### 2.1 Synthesis of TiO_2_/Cu_2_O Heterojunction

All of the chemicals were of analytical grade and were used without additional purification.

For TiO_2_/Cu_2_O preparation, 200 mg of TiO_2_ P25 (Evonik) nanopowder was suspended under sonication with 200 μL of 1.2 M copper­(II) sulfate pentahydrate (CuSO_4_·5H_2_O, Sigma-Aldrich, purity ≥ 98%) solution, serving as the metallic precursor, for 10 min in 100 mL of deionized (DI) water. From this point on, the suspension was heated and maintained at 50 °C, with continuous stirring, for 2 h before proceeding to the next step.

Subsequently, 200 μL of 4.8 M sodium hydroxide (NaOH, Sigma-Aldrich, purity ≥ 99%) solution, used as the precipitating agent, was added dropwise. Finally, after 1 h, 400 μL of 1.2 M ascorbic acid (C_6_H_8_O_6_, Sigma-Aldrich, purity ≥ 99%) solution, acting as the reducing agent (and possibly capping agent^26^), was added and kept under stirring for 30 min. The resulting product was collected and purified by centrifugation (5 min at 5200 rpm), initially using the mother liquor, followed by four washes with DI water and a final rinse with absolute ethanol. After washing, the sample was dried overnight at room temperature in a fume hood. Infrared spectroscopy analysis (Figure S1) confirmed that no ascorbic acid remained adsorbed on the surface of the final product.

### 2.2 Characterization of TiO_2_/Cu_2_O Heterojunction

X-ray powder diffraction (XRPD) analysis was carried out using a Bruker D8 Advance diffractometer with Cu Kα radiation (λ = 1.5418 Å) to identify the phase composition of the sample.

Measurements were taken at room temperature over a 2θ range of 20 to 80°, with a step increment of 0.02° and a collection time of 2 s per step. The weight percentage of the Cu_2_O phase was determined through Rietveld refinement by using TOPAS 4.2 software. Structural data of TiO_2_ phases and Cu_2_O were kept fixed during refinement.

Nitrogen adsorption/desorption experiments were conducted at −196 °C with a NOVAtouch LX2 (Quantachrome) to evaluate textural properties. Before the measurements, the samples were degassed at 120 °C for 2 h. The specific surface area was determined from the adsorption isotherms by using the Brunauer–Emmett–Teller (BET) method. The mesoporous volume was determined from the desorption isotherms using the Barrett–Joyner–Halenda (BJH) approach.

To evaluate the Cu_2_O distribution over TiO_2_, the obtained heterojunction was analyzed by scanning transmission electron microscopy (STEM). Specifically, high-angle annular dark-field (HAADF) imaging and energy dispersive X-ray spectroscopy (EDS) analysis were performed. TEM analysis were carried out using a Thermo Fisher Scientific Talos 200X TEM, operating at 200 kV. For sample preparation, a small quantity of the powder was dispersed in isopropyl alcohol using ultrasonic treatment for 30 min, and then deposited onto a holey carbon film mounted on a copper grid. Additionally, scanning electron microscopy (SEM) using a TESCAN Clara SEM equipped with a Schottky-type Field Emission Gun (FEG) was carried out. For this experiment, a small amount of powder was deposited on a stub with carbon tape.

The reflectance spectra were acquired by diffuse reflectance spectroscopy (DRS) using Spectralon as a reference standard on a PerkinElmer Lambda 650 spectrometer. The sample’s band gap energies (*E*_g_) were estimated by converting DRS data using the Kubelka–Munk function.

Additionally, the photoelectrochemical properties of the as-obtained heterojunction were studied by using electrochemical impedance spectroscopy (EIS) and open-circuit potential (OCP) transients. The tests were conducted with a potentiostat/galvanostat (PGSTAT204, Metrohm) equipped with a standard 3-electrode electrochemical setup and a quartz cell. The working electrode was a glassy carbon electrode (GCE) with an effective surface area of 0.071 cm^2^, while the reference and counter electrodes were a saturated calomel electrode (SCE) and a platinum wire electrode, respectively.

EIS measurements were performed over a frequency range from 1 MHz to 10 mHz, with a 10 mV amplitude sinusoidal wave, and the transient of the OCP was conducted by applying UV-A radiation during 20 s on–off cycles. All tests were conducted using a 0.5 mol L^–1^ Na_2_SO_4_ solution (pH = 7) as the supporting electrolyte. The working electrodes were prepared by polishing with a 0.30 μm alumina powder suspension, followed by sonication in 0.5 mol L^–1^ H_2_SO_4_ for 10 min. Then, 1 mg of each material was added to a mixture of 1 mL of methanol, 0.1 mL of 5% Nafion, and 1.4 mL of distilled water. This suspension was sonicated for 1 h, after which a 20 μL aliquot was applied to the surface of the GCE and dried at room temperature. A 9 W UV-A light (the least energetic ultraviolet light, with wavelengths between 315 and 400 nm) lamp (OSRAM) was used as the light source for the photoelectrochemical tests.

### 2.3 Hydrogen Photoproduction

For the H_2_ photoproduction experiments, 26 mg of the photocatalysts were ultrasonically dispersed in absolute ethanol and added dropwise onto a circular cellulose filter paper (from Albet, thickness 0.18 mm, pore size 35–40 μm, 80 g m^–2^, and a total impregnated area of 11.94 cm^2^). Subsequently, the paper with the photocatalyst was dried in an oven at 50 °C. Once dried, the paper was placed in a tubular glass photoreactor, which was then sealed appropriately. The experiments were conducted at room temperature and atmospheric pressure. The photoreactor’s input consisted of a mixture of argon (used as the carrier gas), water, and ethanol (water/ethanol = 9:1 molar ratio). Argon was bubbled through the saturator containing the water/ethanol mixture to achieve a continuous flow of 20 mL min^–1^. The gas products were monitored online every 4 min using a gas chromatograph (Agilent, Micro-GC 490). Details of the H_2_ photoproduction test apparatus were provided in previous work.^16^ In this study, a spotlight source of a solar light simulator (Hamamatsu Photonics K.K., model L9588–06A) with an irradiance of 60 ± 0.5 mW cm^–2^ was used. The solar-to-hydrogen ratio (η_STH_) was calculated using eq 1

1 $$\eta_{\mathrm{STH}}\left(\%\right)=\frac{\left(n_{H_{2}}\cdot \Delta G\cdot 100\right)}{\left(S\cdot P\right)}$$

where *n*_H_2__ is the produced hydrogen (mmol s^–1^), Δ*G* is the Gibbs Free energy of the water formation (237 kJ mol^–1^), *S* is the irradiated area in the photoreactor (cm^2^), and *P* is the light irradiance (W cm^–2^).^27,28^

## 3 Results and Discussion

Figure 1a presents the H_2_ photoproduction rates for TiO_2_ and the TiO_2_/Cu_2_O heterojunction. As observed, adding Cu_2_O significantly improved the photocatalytic production of hydrogen under sunlight. Strictly speaking, the TiO_2_/Cu_2_O heterojunction achieved an H_2_ photoproduction rate of 8.51 mmol h^–1^ g^–1^, which represents an increase of more than 280 times compared to the production of neat TiO_2_, which yields only 0.03 mmol h^–1^ g^–1^. In addition, cycling tests demonstrated that the TiO_2_/Cu_2_O heterojunction maintained reasonable photostability after several cycles (Figure 1b), with only a slight reduction (∼15%) in its performance, demonstrating its reusability. Although the observed reduction of H_2_ photoproduction after the fifth cycle may be attributed to a partial oxidation of Cu^+^ to Cu^2+^ species,^24^ the deactivation of 15% can be also correlated to the sintering (agglomeration) of Cu_2_O nanoparticles to larger particles, as proposed by Chen et al.^7^ (*vide infra* for XRPD considerations about TiO_2_/Cu_2_O heterojunctions before and after cycling).

**Figure 1:**
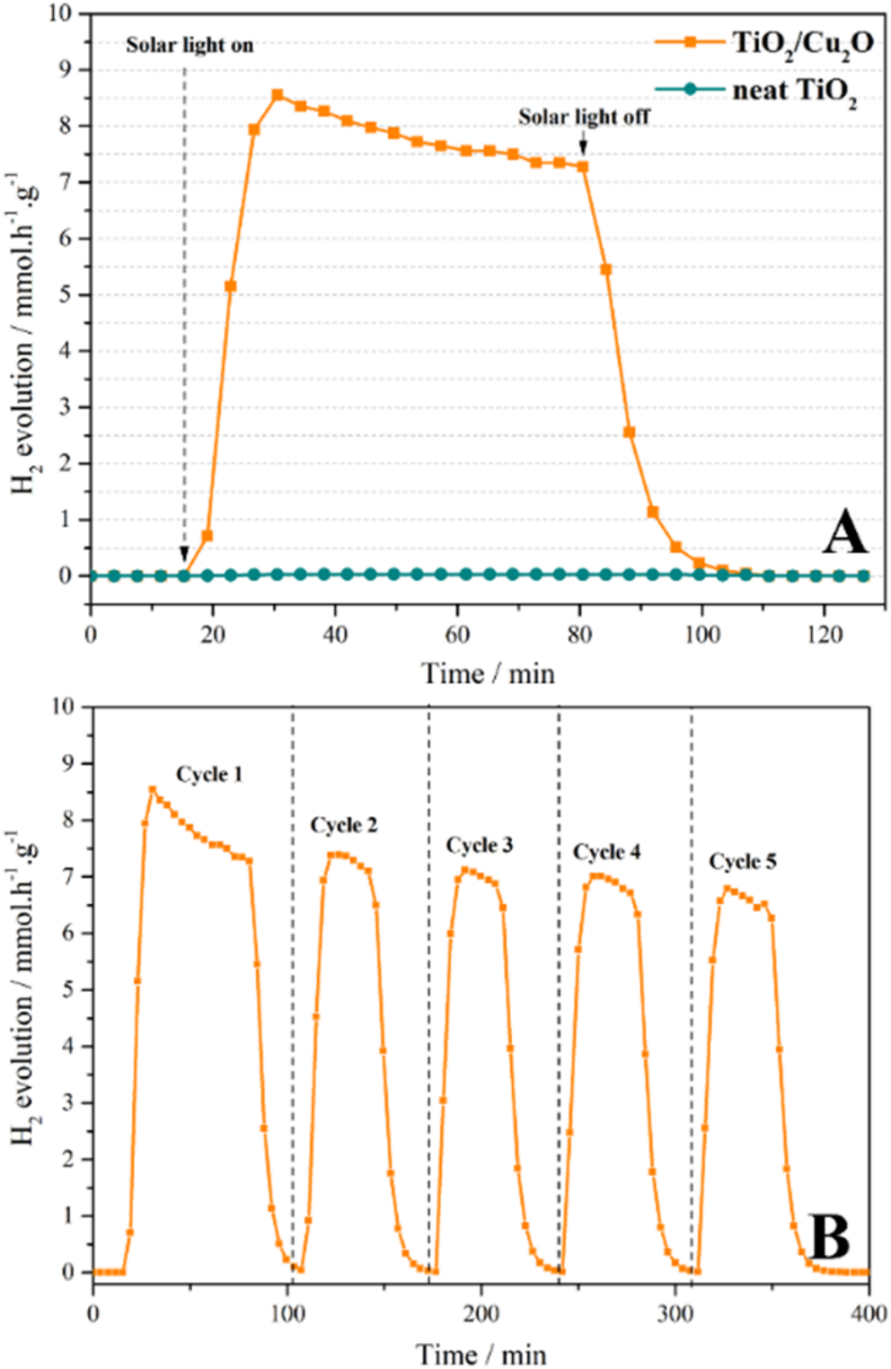
(A) H_2_ photoproduction by the TiO_2_/Cu_2_O heterojunction and TiO_2_ alone; (B) cycling tests of the TiO_2_/Cu_2_O heterojunction.

Furthermore, the η_STH_ value for the TiO_2_/Cu_2_O heterojunction was 2.03%, which is in line with some other photocatalytic systems, ranging between 1 and 3% in η_STH_ values.^4,29^ On the other hand, TiO_2_ alone exhibited an η_STH_ of only 0.01%. The performance of several catalysts in the Cu_*x*_O/TiO_2_ system reported in the literature over the last five years for H_2_ photoproduction is compiled in Table 1. The best way to compare photocatalysts is through the η_STH_ (eq 1) or the apparent quantum efficiency (AQY); therefore, these were the performance parameters plotted in Table 1. Unfortunately, only a few authors reported this data.

**Table 1:** Summary of Cu_*x*_O/TiO_2_-Based Photocatalysts for H_2_ Photoproduction

photocatalyst	light source	H^+^ source solution	η_STH_ (%)	AQY (%)	references
Cu_1.5_-TNTs	solar light	water/glycerol (5% v/v)	--	--	^24^
Cu_2_O/TiO_2_	UV LEDs	water/ethanol [9:1 M]	--	6.4	^21^
TiCu-1	solar light	water/methanol (25% v/v)	--	0.028	^25^
CT–70	Xe lamp	water/methanol [4:1 M]	--	--	^30^
CT-1.5	Xe lamp	water/10 wt % triethanolamine	--	--	^31^
CT-E10	metal halide lamp	water/glycerol (5% v/v)	--	--	^32^
CP-60	Xe lamp	water/methanol (20% v/v)	--	--	^33^
TiO_2_/Cu_2_O	solar light	water/ethanol [9:1 M]	2.03	--	this work

Figure 2a shows the experimental XRPD patterns of the TiO_2_/Cu_2_O and TiO_2_ P25 samples. Regarding the most distinguishable diffraction lines, for the neat TiO_2_ sample, the lines observed at 2θ values of 25.3, 37.9, 48.1, 54, 55.2, and 75.2° correspond to the (101), (004), (200), (105), (211), and (215) crystallographic planes of anatase (PDF 89–4921), respectively. The lines at 2θ values of 27.5, 36.1, 41.3, 62.9, 69.1, and 70° correspond to the (110), (101), (111), (002), (301), and (112) crystallographic planes of rutile (PDF 89–4920).

**Figure 2:**
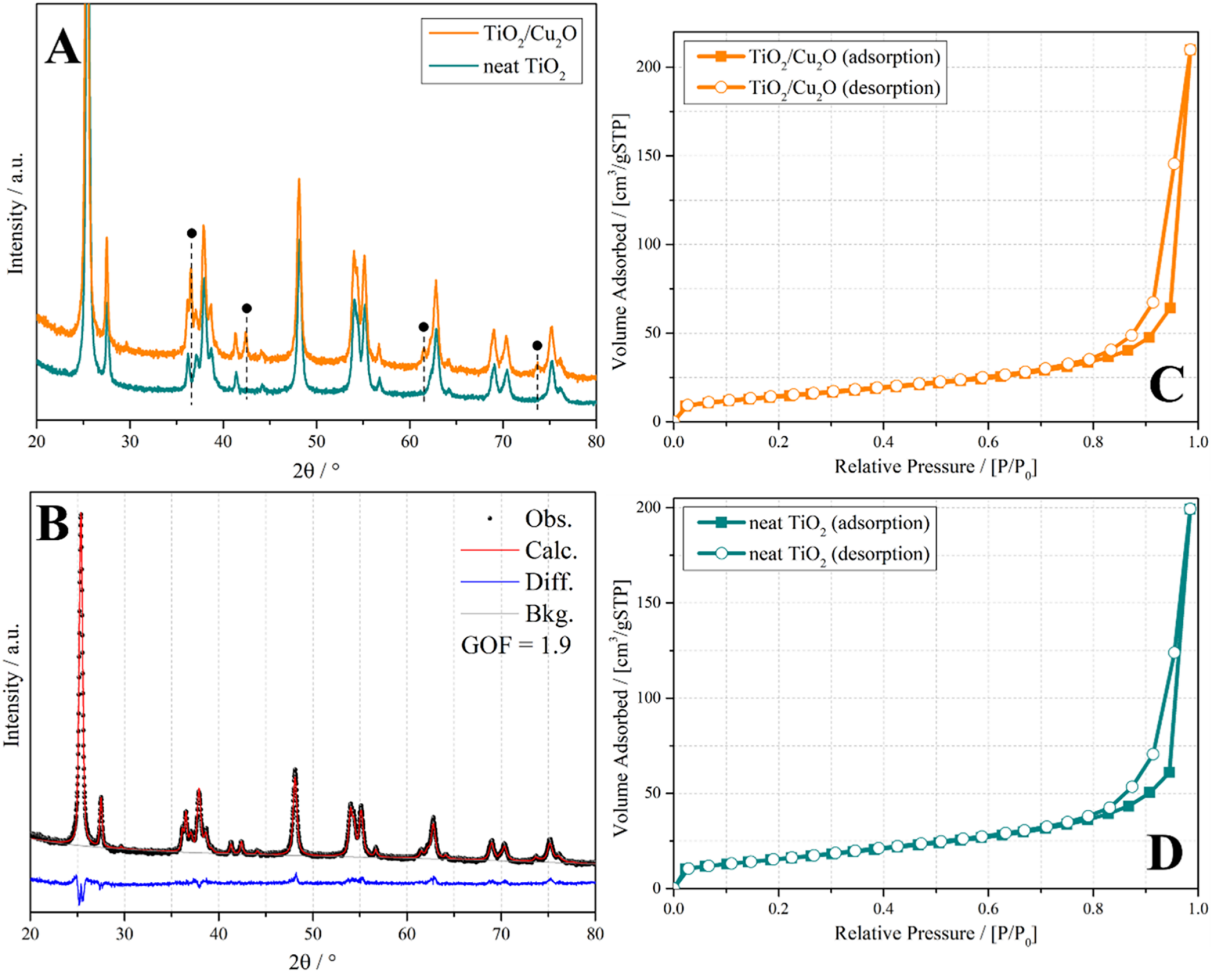
(A) Experimental XRPD patterns of TiO_2_/Cu_2_O heterojunction and TiO_2_ P25, black circles stand for Cu_2_O diffraction lines in the TiO_2_/Cu_2_O pattern; (B) Rietveld refinement of the experimental pattern of TiO_2_/Cu_2_O heterojunction (GOF stands for goodness of fit parameter, attesting good quality of fit); (C) N_2_ adsorption–desorption isotherms of TiO_2_/Cu_2_O heterojunction; and (D) TiO_2_ P25.

In the TiO_2_/Cu_2_O heterojunction, in addition to these same diffraction lines, there are lines situated at 2θ values of 36.5, 42.4, 61.6, and 73.7°, which are ascribed to the (111), (200), (220), and (311) Cu_2_O-cuprite (PDF 77–0199) crystal planes; black circles mark these diffraction lines in Figure 2a. The sharp diffraction lines and low background suggest good crystallinity for both samples. An amount of 3.9 wt % of Cu_2_O was calculated by the quantitative Rietveld method (Figure 2b). The absence of shifts and broadening in the diffraction lines of the TiO_2_ polymorphs in the TiO_2_/Cu_2_O sample, concerning the pure TiO_2_–P25 sample, indicates that there was no doping of Cu into the TiO_2_ structure. This result suggests that Cu fully deposits on the surface of TiO_2_ as the Cu_2_O phase.

The textural characteristics of the samples were studied through the N_2_ adsorption–desorption isotherm analysis. According to the IUPAC classification, the N_2_ adsorption/desorption curves for both neat TiO_2_ and TiO_2_/Cu_2_O heterojunction exhibited a type IVa isotherm (Figure 2c,d), which is indicative of mesoporous materials. The specific surface area of neat TiO_2_ was 56.0 m^2^ g^–1^, while the TiO_2_/Cu_2_O heterojunction exhibited a surface area of 51.6 m^2^ g^–1^. This small difference indicates that the addition of Cu_2_O did not significantly modify the surface area. On the other hand, the pore volume of the TiO_2_/Cu_2_O heterojunction (0.23 cm^3^ g^–1^) was slightly higher than that of neat TiO_2_ (0.19 cm^3^ g^–1^), possibly due to the modification of the porous structure due to the presence of Cu_2_O.

Despite the importance of textural properties for surface phenomena, such as photocatalysis, the minimal differences observed for specific area and total pore volume of the two materials (TiO_2_/Cu_2_O heterojunction and TiO_2_ P25) may suggest that these parameters were not the determining factors for the marked improvement in photocatalytic activity observed in the present study for the TiO_2_/Cu_2_O heterojunction. Therefore, the presence of Cu_2_O was likely crucial for efficient charge separation and enhanced solar light harvesting by the catalyst, thus overcoming the influence of textural properties in this case.

To gain deeper insights into the Cu_2_O distribution over nano-TiO_2_ support (P25), elemental mapping of Ti and Cu using STEM-EDS and STEM-HAADF analyses were employed (Figure 3a,b) to investigate the TiO_2_/Cu_2_O heterojunction (Figure 3c,d). The HAADF is a well-known technique for generating Z-contrast (atomic number contrast) images due to Rutherford scattering.^34^ Due to the configuration of the HAADF detector, which collects quasi elastically scattered electrons, *i.e.*, those that undergo a large deviation in their trajectory after interacting with heavier atoms presented in the sample are preferentially acquired.^35^ This technique, therefore, reveals as brighter the regions with higher atomic numbers.^36^ From the analysis of Figure 3b–d, it can be observed that the TiO_2_/Cu_2_O heterojunction achieved excellent dispersion of the Cu_2_O phase on the TiO_2_ support, a result of the extremely small size of the Cu_2_O particles (some smaller than 2 nm). This excellent Cu_2_O dispersion on the TiO_2_ support was probably crucial in promoting efficient separation of photogenerated charges and minimizing the electronic recombination, which proved to be critical for photocatalytic activity for H_2_ production. The STEM-EDS mapping (Figure 3a) was fundamental in confirming the homogeneity of copper and titanium dispersion as well as demonstrating the integrity of the heterojunction.

**Figure 3:**
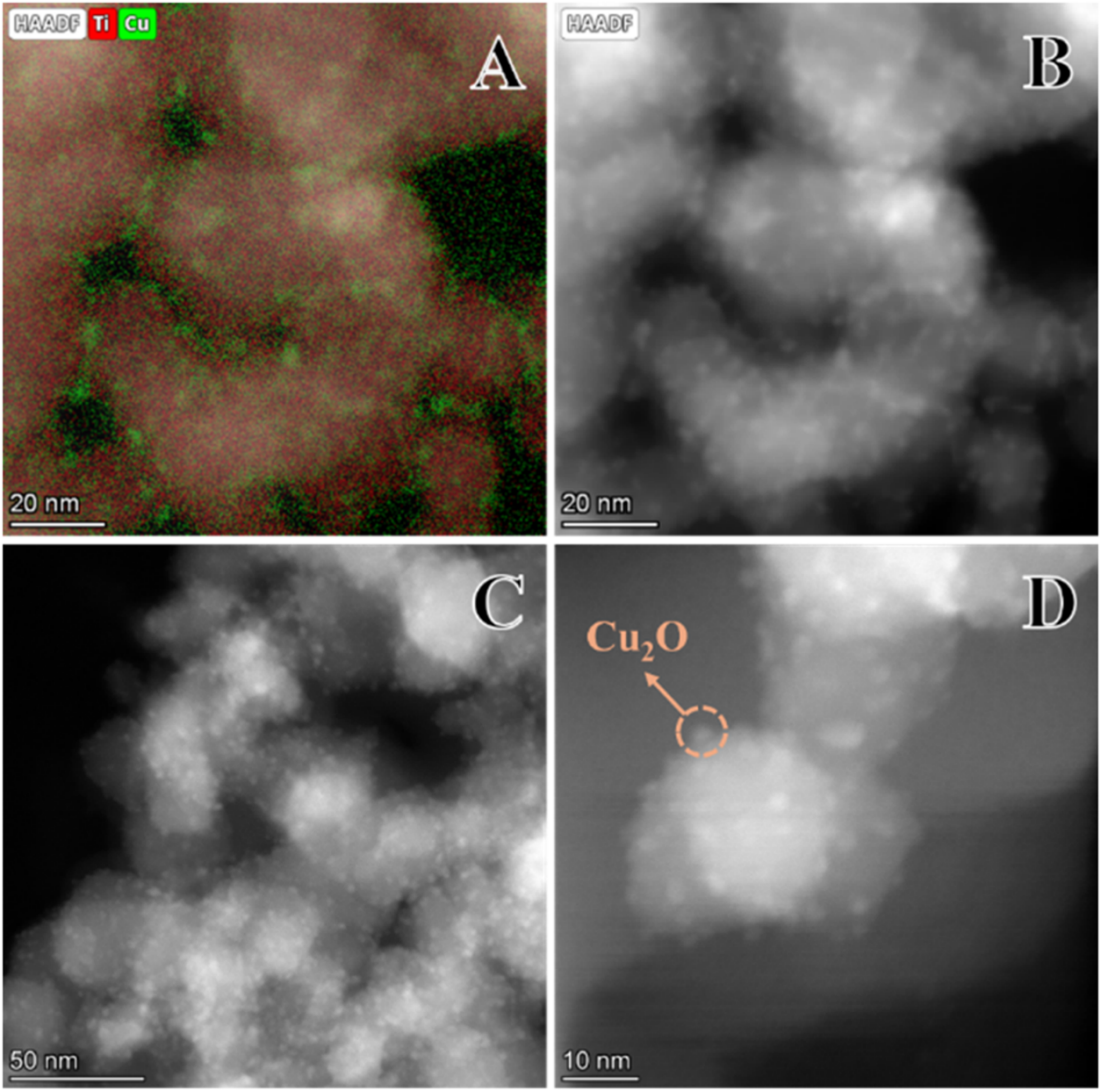
(A) STEM-EDS elemental mapping of titanium and copper; (B) HAADF image of the area mapped by EDS; (C, D) HAADF images of TiO_2_/Cu_2_O heterojunction.

The secondary electron images obtained by SEM (Figure S2a) were collected to probe a broader area of the TiO_2_/Cu_2_O heterostructure. The Ti (Figure S2b) and the O elemental maps (Figure S2c) show a consistent and homogeneous distribution throughout the sample, which was expected since TiO_2_ was used as a support. Notably, the Cu elemental map (Figure S2d) also reveals a uniform distribution, confirming that the copper phase, namely, Cu_2_O, is mostly well-dispersed on the surface of TiO_2_ rather than as segregated large particles or agglomerates.

These findings, together with the XRPD results (Figure 2A, B), suggest that the synthesis method reported here allows for uniform deposition of Cu_2_O on the TiO_2_ support, instead of Cu doping in the TiO_2_ lattice. This feature is crucial for forming an efficient p-n interface as it maximizes the interface between the phases that make up the heterojunction.^4^ This partly explains the outstanding photocatalytic performance observed for the TiO_2_/Cu_2_O sample compared to TiO_2_ P25. However, interestingly, the diffraction lines of the Cu_2_O phase were relatively sharp (Figure 2a), while for such small particle sizes (some even smaller than 2 nm) as observed in the STEM analyses (Figure 3), much broader diffraction lines would be expected, in accordance with X-ray powder diffraction theory. This, therefore, suggests the presence of larger Cu_2_O particles segregated in some regions of the sample (not detected in the STEM analysis). The addition of Cu may have led to the formation of ultrasmall Cu_2_O nanoparticles (<2 nm), well-dispersed in regions where the TiO_2_ support was available, while the excess copper could have agglomerated into larger particles in other areas. This agglomeration of copper would explain the sharp diffraction lines of Cu_2_O observed in XRPD (Figure 2A), which are inconsistent with the small particles detected in the STEM analysis. In addition, 15% deactivation of H_2_ photoproduction after the fifth cycle (Figure 1B) cannot be justified by XRPD data. XRPD pattern of the heterojunction after cycling (Figure S3) does not indicate any appreciable change in comparison to the XRPD pattern of TiO_2_/Cu_2_O heterojunction before cycling tests (Figure 2A, B). The diffraction lines of Cu_2_O are still the only diffraction lines related to some copper phase. However, the XRPD pattern (Figure S3) possibly is not able to illustrate eventual changes of the Cu_2_O nanoparticles (≤2 nm) dispersed over the TiO_2_ support.

The ultraviolet–visible (UV–vis) absorption spectra of the TiO_2_/Cu_2_O heterojunction and neat TiO_2_, presented in Figure 4a, reveal that the incorporation of Cu_2_O onto the TiO_2_ support broadened the light absorption, including visible light absorption (between 400 and 700 nm), absent in neat TiO_2_. The analysis of the optical band gap (*E*_g_) was performed using reflectance data as input, converted to absorbance and further to Tauc plot by the Kubelka–Munk function. The resulting Tauc plots are shown in Figure 4b. TiO_2_ exhibited an *E*_g_ = 3.2 eV, attributed to anatase,^22^ the dominant polymorph of TiO_2_ in P25.

**Figure 4:**
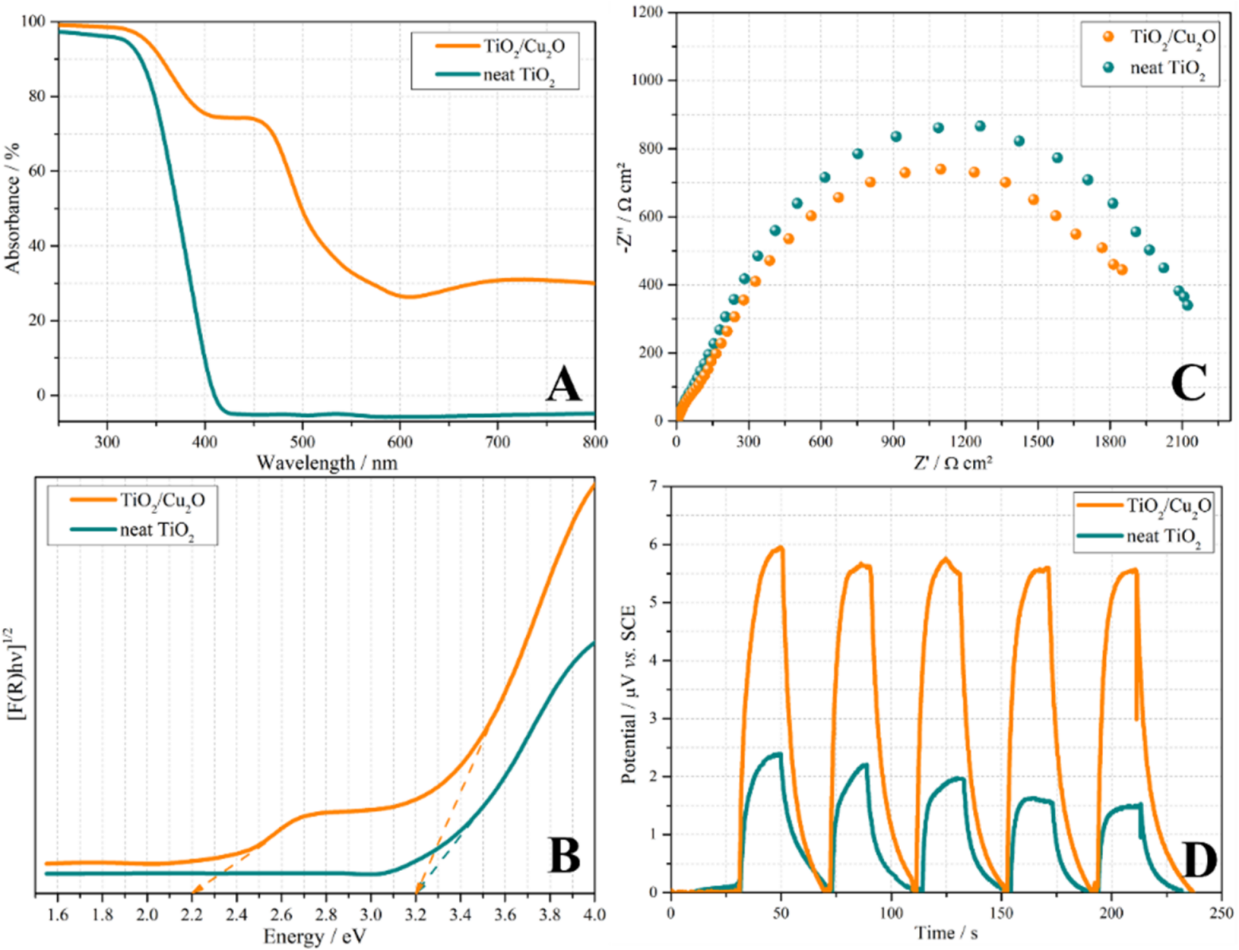
(A) Absorbance spectra; (B) Tauc plots of (*F*(*R*)*hv*)^1/2^ *vs* energy of TiO_2_/Cu_2_O heterojunction and TiO_2_ P25; (C) Nyquist plots of TiO_2_/Cu_2_O heterojunction and TiO_2_ P25 under UV-A radiation; and (D) Photovoltage of TiO_2_/Cu_2_O heterojunction and neat TiO_2_ P25 in the presence and absence of UV-A radiation.

On the other hand, the TiO_2_/Cu_2_O sample exhibited, in addition to the *E*_g_ of anatase, an *E*_g_ = 2.2 eV, in accordance with the reported value in the literature for Cu_2_O.^37^ These results demonstrate that the greater efficiency in utilizing the solar spectrum achieved by the TiO_2_/Cu_2_O heterojunction, extending light absorption into the visible range, resulted in markedly superior photocatalytic H_2_ production.

Figure 4c shows the Nyquist diagrams of pure TiO_2_ and TiO_2_/Cu_2_O under UV-A light. As shown, the TiO_2_/Cu_2_O heterojunction presents a smaller capacitive arc. Specifically, the transfer resistance (*R*_ct_) values obtained from the electrochemical circuit (Figure S4) were 2478 and 2250 Ω cm^2^ for TiO_2_ alone and the TiO_2_/Cu_2_O heterojunction, respectively. These *R*_ct_ values indicate that under the studied conditions, the TiO_2_/Cu_2_O heterojunction has the lowest *R*_ct_ and consequently better photoelectrochemical properties, such as a higher charge carriers’ lifetime.^4^

The normalized open-circuit potential (OCP) transients for pure TiO_2_ and the TiO_2_/Cu_2_O heterojunction were measured with alternating equal dark and light intervals of 20 s (Figure 4d). For both materials, the presence of radiation caused an increase in potential, which is related to the generation of photocatalytic charge carriers.^38^ However, the TiO_2_/Cu_2_O heterojunction exhibited a potential variation approximately three times higher than that of TiO_2_ P25, demonstrating that the addition of Cu_2_O boosted the photocatalytic activity of the heterojunction. Additionally, when evaluating the five on–off cycles, it is evident that for pure TiO_2_ there is a decrease in potential throughout the cycles, indicating recombination between the hole–electron pairs, and consequently, a decrease in the photocatalytic properties of the material.^38,39^

The photoelectrochemical results (Figure 4c,d) indicated that the TiO_2_/Cu_2_O heterojunction prepared by the synthesis method presented herein was beneficial for the heterojunction’s photoelectrochemical properties, resulting in greater photocatalytic activity and reduced recombination, corroborating the photocatalytic H_2_ production results presented in Figure 1.

## 4 Conclusions

This study found that it is possible, at least partially, to disperse Cu_2_O into nanometric species, as small as 2 nm, over a nano-TiO_2_ support, *via* a simple, one-stage method, free of common capping or stabilizing agents, carried out under low temperature (50 °C) and at ambient pressure. The as-prepared heterojunction exhibited a remarkable improvement in photocatalytic hydrogen production under sunlight, being 280 times more efficient than TiO_2_ alone.

The STEM (HAADF and EDS) analyses revealed excellent dispersion of ultrasmall Cu_2_O nanoparticles (even smaller than 2 nm) on the surface of TiO_2_, confirming the formation of a predominantly homogeneous heterojunction. However, sharp diffraction lines for the Cu_2_O phase were observed in the XRPD analysis, revealing the possible presence of segregated regions with larger Cu_2_O particles. This finding may indicate that the copper content still exceeded the spatial dispersion capacity of this phase on the TiO_2_ support. In addition, DRS and EIS measurements indicated a significant extension of light absorption to the visible region and a lower recombination rate of e^–^/h^+^ pairs in the TiO_2_/Cu_2_O heterojunction, respectively.

In summary, our findings demonstrate the applicability and effectiveness of a soft-chemistry synthesis route for preparation of TiO_2_/Cu_2_O heterojunctions, eliminating the need for addition of a common capping/stabilizing agent (apparently, reducing agent AA, and its byproducts, in alkaline environment may exercise this role, too) and still achieves remarkable dispersion of the Cu_2_O phase with ultrasmall particles on the nano-TiO_2_ support, promoting better solar spectrum utilization and extending the lifetime of photogenerated charge carriers. It is suggested that this new material shows great potential as an economically viable catalyst for sustainable H_2_ production driven by solar energy.

## Supplementary Material



## Notes

Supporting Figures: S1- Infrared spectroscopy analysis; S2- SEM image of TiO2/Cu2O heterojunction; S3- XRPD pattern after cycling, and S4- EIS and equivalent circuit (PDF)
