# Supplementary material for: TiO2/Cu2O Heterojunction with Ultrafine Cu2O Dispersion and Enhanced Performance for Solar-Driven Hydrogen Production: A Low-Temperature, Ambient Pressure, and Common Stabilizing Agent-Free Synthesis Approach
Source: ACS Omega. 2025 Aug 27;10(35):39814–22. doi: 10.1021/acsomega.5c03805 (PMC12423826; doi:10.1021/acsomega.5c03805)
Supplement: Supplementary file 1 [file ao5c03805_si_001.pdf]

Supporting Information

TiO_2/Cu_2O heterojunction with ultrafine Cu_2O dispersion and enhanced performance for solar-driven hydrogen production: a low-temperature, ambient pressure, and common stabilizing agent-free synthesis approach

Emanuel do Couto-Pessanha[a,b], Victor Magno Paiva[c], Marianne Diniz[a], Eliane D'Elia[c], Khrissy Aracélly Reis Medeiros[a], Jordi Llorca[b], Bojan A. Marinkovic[a,]*

[a]*Department of Chemical and Materials Engineering, Pontifical Catholic University of Rio de Janeiro (PUC-Rio), 22453-900, Rio de Janeiro, RJ, Brazil*

[b]*Institute of Energy Technologies, Department of Chemical Engineering and Barcelona Research Center in Multiscale Science and Engineering, Universitat Politècnica de Catalunya, EEBE, Eduard Maristany 16, 08019 Barcelona, Spain*

[c]*Institute of Chemistry, Federal University of Rio de Janeiro, UFRJ, 21941-909, Rio de Janeiro, Brazil*

*Author to whom correspondence should be addressed: bojan@puc-rio.br (Bojan A. Marinkovic)

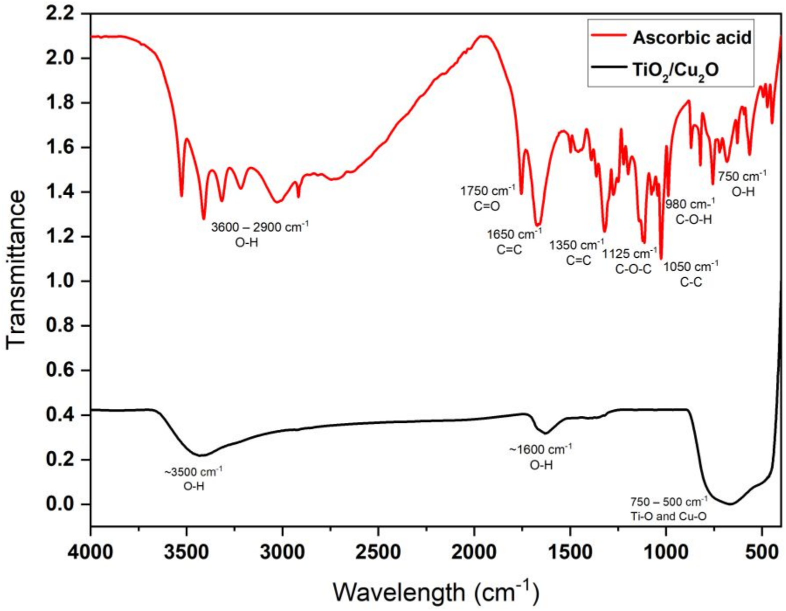

Figure S1. Infrared spectroscopy analysis.

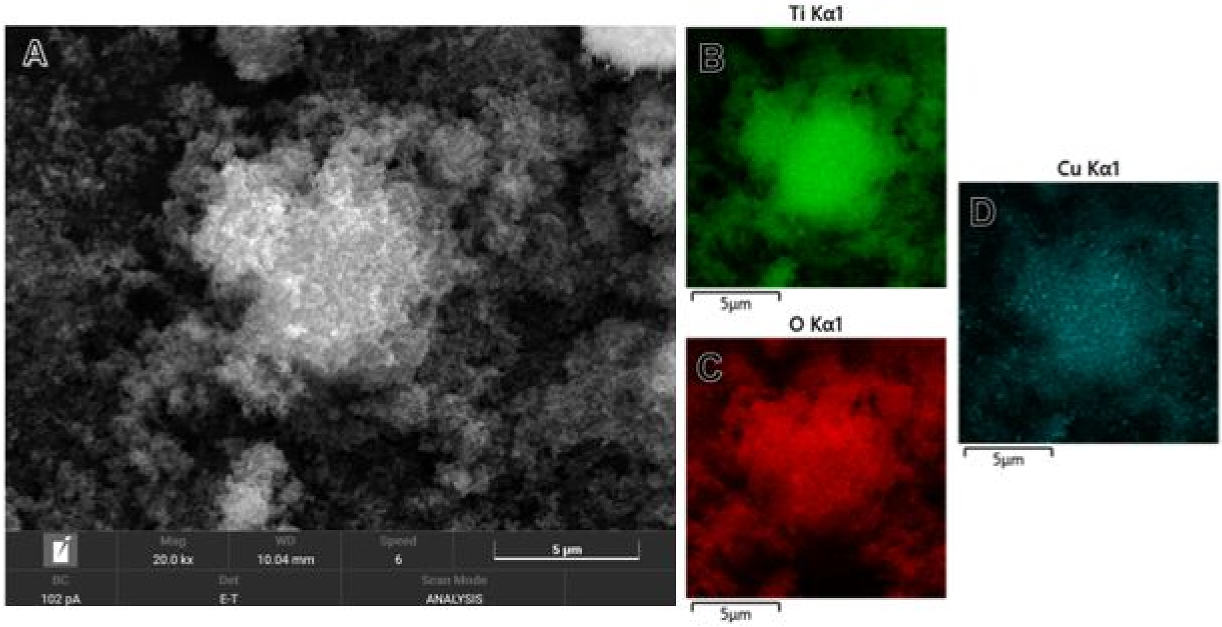

Figure S2. (a) Secondary Electron-SEM image of TiO_2/Cu_2O heterojunction, and the area mapped by EDS (b) titanium; (c) oxygen, and (d) copper elemental maps.

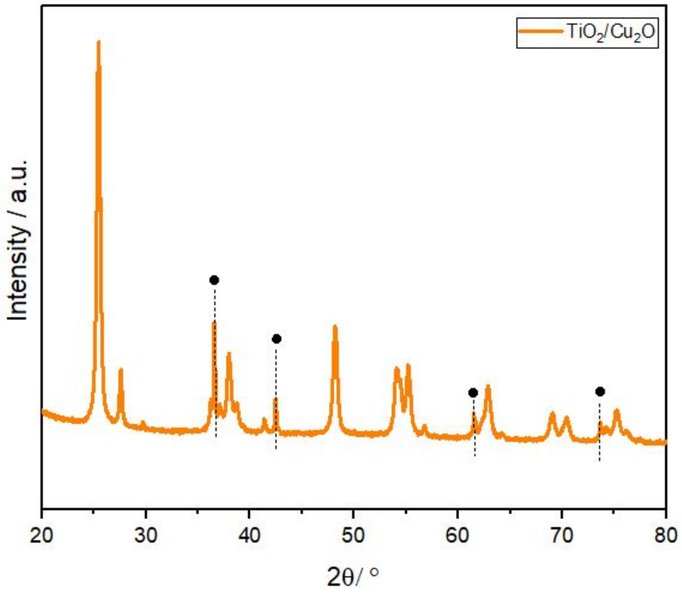

Figure S3. XRPD pattern after cycling.

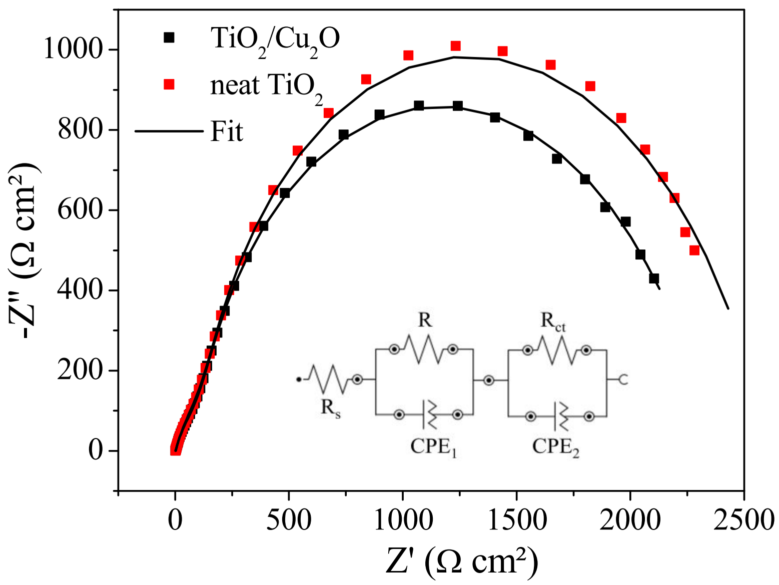

Figure S4. EIS and equivalent circuit employed to fit experimental data (which R_s is the electrolyte resistance, R and CPE_1 represent the bulk material impedance of Nafion film, R_{ct} and CPE_2 represent the GCE interface's charge transfer resistance and constant phase element).
